# Not Tuberculosis After All: The “Hare‐Raising Case” of Tularemia in a Patient With Advanced HIV Infection

**DOI:** 10.1155/crdi/6697292

**Published:** 2026-06-28

**Authors:** J. X. Geerdink, B. J. Van Welzen

**Affiliations:** ^1^ Department of Infectious Diseases, University Medical Center Utrecht, Utrecht, the Netherlands, umcutrecht.nl; ^2^ Department of Internal Medicine, Canisius Wilhelmina Ziekenhuis, Nijmegen, the Netherlands

**Keywords:** AIDS, *Francisella tularensis*, *Francisella tularensis holarctica*, HIV, immunocompromised patients, tularemia

## Abstract

We report a rare case of pneumonic tularemia in a person with an advanced HIV infection, in which a mediastinoscopy was ultimately required for microbiological confirmation. Serological testing remained negative. This case illustrates the extent to which pneumonic tularemia can mimic pulmonary tuberculosis and illustrates the limitations of serology in immunocompromised individuals. Clinicians should consider tularemia in the differential diagnosis of pulmonary disease with necrotizing lymphadenopathy, even in regions where the incidence is low.

## 1. Introduction

Tularemia is a rare zoonotic disease caused by *Francisella tularensis*, a facultative intracellular, Gram‐negative bacterium. Human transmission typically occurs through contact with infected animals, arthropod bites, or environmental exposures. The pathogen is highly virulent, with an inoculum as few as 10–50 organisms being sufficient to cause infections [1]. In the Netherlands, tularemia is colloquially referred to as “hare’s plague,” reflecting its association with hunting and handling of wild lagomorphs. Globally, *F. tularensis* has distinct subspecies with geographical variations: the highly virulent *tularensis* subspecies in North America and the less virulent but more widespread *holarctica* subspecies in Europe [2]. Autochthonous infection in the Netherlands with *Francisella tularensis holarctica* is very rare with only 26 cases described between 2011 and 2021 [3].

Tularemia presents in six distinct clinical syndromes: ulceroglandular, glandular, oculoglandular, pharyngeal, pneumonic, and typhoidal, depending largely on the route of infection. Pneumonic tularemia is uncommon and may result from inhalation or hematogenous spread. The clinical picture is nonspecific, with fever and respiratory symptoms. Radiological studies usually show pulmonary infiltrates accompanied with hilar and/or mediastinal lymphadenopathy; pleural effusion is not uncommon [4, 5]. The diagnosis can be challenging due to the fastidious growth characteristics of *F. tularensis*, with standard cultures demonstrating low sensitivity. Serological testing remains the mainstay for diagnosis but requires clinical alertness.

## 2. Case Report

A 24‐year‐old female from Kenya, with no significant medical history, was admitted to hospital because of persistent diarrhea and fever for two weeks. Further history taking revealed unintended weight loss (> 10%) over the past few months. At admission, she had been living in the Netherlands for 8 months, working as an au pair. There was no recent travel history or animal exposure. Physical examination revealed no abnormalities; laboratory studies showed a microcytic anemia and mildly elevated inflammatory markers (Table 1). Fecal polymerase chain reaction (PCR) was positive for *Campylobacter jejuni*, and a 3‐day course of azithromycin (500 mg once daily) was initiated, with a good clinical response. Blood cultures remained negative.

**TABLE 1:** Laboratory results on admission (October 28, 2023).

Parameter	Result	Reference range	Unit
*Hematology*			
Hemoglobulin	4.8	7.5–10.0	mmol/L
Hematocrit	0.25	0.35–0.45	L/L
Erythrocytes	3.49 × 10^12^	4.00–5.00 × 10^12^	/L
Mean corpuscular volume (MCV)	72	80–98	fL
Mean corpuscular hemoglobin (MCH)	1375	1700–2100	amol
Red cell distribution width (RDW)	17.1	11.5–14.0	%
Thrombocytes	257 × 10^9^	150–450 × 10^9^	/L
Leukocytes	3.5 × 10^9^	4.0–11.0 × 10^9^	/L
Neutrophils	2.7 × 10^9^	1.5–8.0 × 10^9^	/L
Lymphocytes	0.42 × 10^9^	1.0–4.0 × 10^9^	/L
Monocytes	0.42 × 10^9^	0.30–1.0 × 10^9^	/L
Eosinophils	< 0.10 × 10^9^	0–0.5 × 10^9^	/L
Basophils	< 0.10 × 10^9^	0–0.2 × 10^9^	/L

*Chemistry*			
CRP	77	< 5.0	mg/L
Sodium	131	136–145	mmol/L
Potassium	3.3	3.5–5.0	mmol/L
Calcium	2.16	2.10–2.55	mmol/L
Urea	3.2	2.5–6.7	mmol/L
Creatinine	63	50–95	μmol/L
eGFR (CKD‐EPI)	> 90	> 60	mL/min/1.73 m^2^
Total bilirubin	17	< 20	μmol/L
Direct bilirubin	6	< 9	μmol/L
Alkaline phosphatase (ALP)	61	30–120	U/L
Gamma‐glutamyl transferase (GGT)	17	< 27	U/L
Aspartate aminotransferase (AST)	39	< 30	U/L
Alanine aminotransferase (ALT)	16	< 35	U/L
Lactate dehydrogenase (LDH)	422	< 250	U/L
Lipase	64	< 56	U/L
Glucose	5.0	3.3–7.8	mmol/L
Ferritin	1690	15–204	μg/L
Haptoglobin	3.8	0.3–2.5	g/L
Albumin	36	35–50	g/L
Vitamin B12	529	140–640	pmol/L
Folate	2.6	7.8–45.3	nmol/L

*Note:* Laboratory findings obtained at hospital admission, prior to the establishment of the final diagnosis. The table summarizes the key hematological, biochemical, inflammatory, and coagulation parameters that contributed to the initial clinical evaluation and diagnostic work‐up.

Because of the unintended weight loss, serological testing for HIV was performed, which came back positive. Additional studies revealed advanced disease: CD4+ lymphocyte count was 9 cells/mm3 and HIV viral load (VL) was 7.04^4 copies/mL. A total body computed tomography showed necrotizing mediastinal lymphadenopathy with limited perihilar consolidation, raising the suspicion for active tuberculosis (Figure 1A). PCR and microscopy on bronchoalveolar lavage samples were negative for *Mycobacterium tuberculosis* (MTB). An interferon‐gamma release assay (Quantiferon) also yielded a negative result.

**FIGURE 1:**
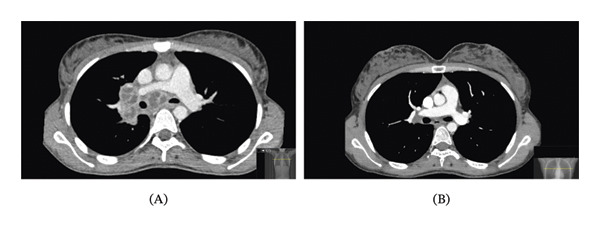
Thoracic computed tomography. (A) Pretreatment CT: Baseline contrast‐enhanced computed tomography (CT) of the thorax at admission shows extensive necrotizing mediastinal lymphadenopathy. (B) Posttreatment CT: Follow‐up CT scan, obtained 3 months after the initiation of antimicrobial therapy with ciprofloxacin, demonstrates complete radiological resolution of the lymphadenopathy.

A high suspicion for tuberculosis persisted given the radiological findings, clinical presentation, and the patient’s origin from a tuberculosis‐endemic region. This suspicion remained despite initial negative results. Additional endobronchial‐guided fine‐needle aspiration of the lymphadenopathy was performed, with MTB PCR being negative and nonconclusive cytology. Empirical antituberculosis therapy was considered, but after extensive deliberation, it was decided to proceed with anterior mediastinoscopy for the collection of cultures and histology. Surprisingly, the culture showed *F. tularensis* subspecies *holarctica,* confirming the diagnosis of tularemia. Susceptibility testing of the isolate yielded minimum inhibitory concentrations (MICs) for azithromycin 0.38 μg/mL, ciprofloxacin 0.032 μg/mL, gentamicin 1.5 μg/mL, and tetracycline 2 μg/mL.

Following these findings, the patient was started on a 14‐day course of oral ciprofloxacin (750‐mg bid). Concurrently, combination antiretroviral therapy (cART), consisting of dolutegravir, emtricitabine and tenofovir disoproxil, was initiated. Patient’s condition improved rapidly, and she could be discharged 2 days after the initiation of ciprofloxacin. Subsequent follow‐up assessments demonstrated radiological resolution of lymphadenopathy and partial immunological recovery (Figure 1B), with the CD4+ lymphocyte count increasing to 80 cells/mm^3^ after three months, and HIV VL decreased to < 50 copies/mL. Serological testing for *F. tularensis* was negative at diagnosis and also at the 3‐month follow‐up. No relapse of tularemia was observed after treatment. The patient was unfortunately temporarily lost to follow‐up while seen at another hospital. However, when reassessed at our center approximately 18 months after initial presentation, there were no clinical or laboratory indications of recurrent or persistent tularemia.

## 3. Discussion

This case highlights several important and unusual features. First, it illustrates the extent to which pneumonic tularemia can mimic pulmonary tuberculosis. In our case, tuberculosis was considered to be the most likely diagnosis based on patient’s region of origin and her severely immunocompromised status. Pneumonic tularemia was deemed unlikely in view of its low incidence in the Netherlands and no known exposure to lagomorphs [6]. Ultimately, mediastinoscopy was pursued to obtain material for a microbiological diagnosis and susceptibility testing, considering the potential toxicity from empirical anti‐TB treatment. Although not routinely performed, invasive diagnostic procedures such as thoraco‐ or mediastinoscopy have been reported in up to 20% of the confirmed pneumonic tularemia cases in Finland [4].

Second, the immunocompromised status of the patient adds a unique element. Data on tularemia in immunocompromised individuals are sparse, with only 18 cases being described to date [7, 8]. While it is generally assumed that immunocompromised hosts are at risk for more severe disease, it is unclear which groups are at the highest risk. Considering the immune response against *F. tularensis* is depended on both cellular and humoral immunity. To our knowledge, only two case reports describe tularemia in individuals with advanced HIV infection. The first report describes a 50‐year‐old male (CD4 220 cells/mm^3^) with community‐acquired pneumonia treated with a 10‐day course of oral levofloxacin [9].

Three weeks after discharge, mycobacterial blood cultures grew small Gram‐negative rods identified as *F. tularensis*, but no further treatment was required as he had recovered. The second report involved a 12‐year‐old boy with severe immunosuppression (CD4 0 cells/mm^3^) presenting with fever, headache, cough, and hepatosplenomegaly [10]. Blood cultures confirmed typhoidal tularemia, and despite appropriate therapy, he experienced two relapses before eventual cure. Notably, serologic tests remained negative in that case.

This leads to our final clinically relevant point: the possibility of false‐negative serology in immunocompromised individuals. While *F. tularensis* serology is generally considered both sensitive and specific [11], our patient’s results remained negative throughout the disease course. This may be attributed to profound immunosuppression, as antibody production can be impaired in advanced HIV‐related immunodeficiency as described in the case above. In addition, our patient had received azithromycin early in the disease course, which may have hindered seroconversion. Early antibiotic therapy has been shown to interfere with antibody development in other infections [12]; although these phenomena is not well established for tularemia, this mechanism does warrant consideration.

Azithromycin is not among the standard treatment options for tularemia, for which aminoglycosides, fluoroquinolones, and tetracyclines are preferred [13]. Macrolides are only used in very specific situations such as pregnancy [14]. In our case, the MIC for azithromycin was low, suggesting possible in vivo activity, but once the diagnosis was confirmed, standard fluoroquinolone therapy was initiated.

In conclusion, we report a rare case of pneumonic tularemia in an individual with advanced HIV infection. This case highlights the importance of considering atypical pathogens in immunocompromised hosts. It illustrates three key clinical points. First, pneumonic tularemia can mimic pulmonary tuberculosis. Second, immunocompromised patients may present with atypical features and more severe disease. Third, serology may be negative, especially with immunosuppression or early antibiotic therapy. In selected cases, invasive procedures may be necessary to confirm the diagnosis and guide appropriate therapy.

## Author Contributions

J. X. Geerdink and B. J. Van Welzen contributed equally to the conception and design of the study, conducted the literature review, and drafted the manuscript. Both authors critically revised the content for important intellectual content.

## Funding

This research received no external funding.

## Disclosure

All authors have approved the final version of the manuscript.

## Ethics Statement

This study was conducted in accordance with institutional and ethical guidelines.

## Consent

The patient provided informed consent for the use of clinical data and images for research and publication purposes. All potentially identifying information has been removed to ensure patient anonymity.

## Conflicts of Interest

The authors declare no conflicts of interest.

## Data Availability

The data that support the findings of this study are available on request from the corresponding author. The data are not publicly available due to privacy or ethical restrictions.
